# Evaluation of the novel TSPO radiotracer [^18^F] VUIIS1008 in a preclinical model of cerebral ischemia in rats

**DOI:** 10.1186/s13550-017-0343-7

**Published:** 2017-11-25

**Authors:** Krishna R. Pulagam, Lorena Colás, Daniel Padro, Sandra Plaza-García, Vanessa Gómez-Vallejo, Makoto Higuchi, Jordi Llop, Abraham Martín

**Affiliations:** 1 Radiochemistry and Nuclear Imaging, Molecular Imaging Unit, CIC biomaGUNE, P° Miramon 182, San Sebastian, Spain; 2 Experimental Molecular Imaging, Molecular Imaging Unit, CIC biomaGUNE, P° Miramon 182, San Sebastian, Spain; 3 Magnetic Resonance Imaging, Molecular Imaging Unit, CIC biomaGUNE, P° Miramon 182, San Sebastian, Spain; 4 National Institutes for Quantum and Radiological Science and Technology, National Institute of Radiological Sciences, Chiba, Japan

**Keywords:** T_2_W-MRI, [^18^F] VUIIS1008, [^18^F] DPA-714, PET, Cerebral ischemia

## Abstract

**Background:**

In vivo positron-emission tomography (PET) imaging of transporter protein (TSPO) expression is an attractive and indispensable tool for the diagnosis and therapy evaluation of neuroinflammation after cerebral ischemia. Despite several radiotracers have shown an excellent capacity to image neuroinflammation, novel radiotracers such as [^18^F] VUIIS1008 have shown promising properties to visualize and quantify the in vivo expression of TSPO.

**Methods:**

Longitudinal in vivo magnetic resonance (MRI) and PET imaging studies with the novel TSPO radiotracer 2-(5,7-diethyl-2-(4-(2-[^18^F] fluoroethoxy) phenyl) pyrazolo [1,5-a] pyrimidin-3-yl)-N, N-diethylacetamide ([^18^F] VUIIS1008), and (N, N-diethyl-2-(2-[4-(2-fluoroethoxy)-phenyl]-5,7-dimethyl-pyrazolo [1,5-a] yrimidin-3-yl)-acetamide ([^18^F] DPA-714) were carried out before and at days 1, 3, 7, 14, 21, and 28 following the transient middle cerebral artery occlusion (MCAO) in rats.

**Results:**

MRI images showed the extension and evolution of the brain infarction after ischemic stroke in rats. PET imaging with [^18^F] VUIIS1008 and [^18^F] DPA714 showed a progressive increase in the ischemic brain hemisphere during the first week, peaking at day 7 and followed by a decline from days 14 to 28 after cerebral ischemia. [^18^F] DPA714 uptake showed a mild uptake increase compared to [^18^F] VUIIS1008 in TSPO-rich ischemic brain regions. In vivo [^18^F] VUIIS1008 binding displacement with VUIIS1008 was more efficient than DPA714. Finally, immunohistochemistry confirmed a high expression of TSPO in microglial cells at day 7 after the MCAO in rats.

**Conclusions:**

Altogether, these results suggest that [^18^F] VUIIS1008 could become a valuable tool for the diagnosis and treatment evaluation of neuroinflammation following ischemic stroke.

## Background

Cerebral ischemia induces the death of ischemic neurons and especially the release of necrotic debris that triggers the inflammatory reaction resulting in a strong activation of the resident glial cells and leucocyte infiltration [1]. As the damage progresses, microglia undergo progressive changes, including altered expression of cell surface markers and inflammation-related genes, process retraction and the acquisition of an ameboid morphology, cell body migration, and increasing phagocytic ability [2]. The inflammatory reaction promotes a dramatic increase of a mitochondrial transmembrane protein, the translocator protein (TSPO), considered as the hallmark of neuroinflammation in brain diseases [3]. Despite this, TSPO overexpression mainly reflects microglia, infiltrated macrophages, and astrocytes activation and proliferation rather than neuroinflammation in its broader sense [4]. Nevertheless, over the last decade, TSPO has become an attractive target for positron-emission tomography (PET) imaging of cerebral inflammation due to its high expression after pathologic situations despite the low expression showed in the healthy cerebral parenchyma [5]. TSPO expression in a wide diversity of neurologic and neurodegenerative diseases has been monitored by PET with a large variety of first generation ([^11^C]*(R)*-PK11195) [6] or second and third generation of radioligands such as [^11^C] SSR180575 [7], [^18^F] PBR28 [8, 9], [^11^C] DAA1106 [10–12], [^11^C] CLINME [13], [^18^F] FEAC [14], [^18^F] FEDAC [14], [^18^F] FEPPA [15, 16], [^18^F] DPA-714 [3, 17, 18], [^18^F] PBR111 [8], [^18^F] PBR06 [19, 20], and [^18^F] GE-180 [21–23], among others, evidencing the interest aroused by TSPO imaging from the bench to the bedside. In fact, the second and third generations of TSPO radioligands have been synthesized to improve the limitations presented by [^11^C] PK11195, including the high level of non-specific binding and the poor signal-to-noise ratio [3]. In particular, [^18^F] DPA-714 and [^18^F] GE-180 have been directly compared with [^11^C] PK11195 after cerebral ischemia in rats confirming the better signal-to-noise ratio in the ischemic lesion and the low level of non-specific binding in the contralateral brain hemisphere [3, 22, 24]. In this sense, a novel TSPO radioligand, 2-(5,7-diethyl-2-(4-(2-[^18^F] fluoroethoxy) phenyl) pyrazolo [1,5-a] pyrimidin-3-yl)-*N*, *N*-diethylacetamide ([^18^F] VUIIS1008), has shown a 36-fold enhancement in binding activity (Ki = 0.3 nM) compared to its parent compound, DPA-714 (Ki = 10.9 nM) [25, 26]. The purpose of the present study was to investigate the usefulness of the novel radiotracer [^18^F] VUIIS1008 to monitor TSPO expression following a preclinical model of cerebral ischemia. For this reason, ischemic rats were subjected to PET studies with [^18^F] VUIIS1008 and [^18^F] DPA-714 from 1 to 28 days after the experimental middle cerebral artery (MCA) occlusion in rats. PET was conducted in parallel with immunohistochemistry of TSPO. The results reported here are particularly relevant for providing novel information on the suitability of [^18^F] VUIIS1008 for TSPO imaging after neurologic and neurodegenerative diseases.

## Methods

### Cerebral ischemia

Adult male Sprague-Dawley rats (296+/− 9, 11 g body weight; Janvier, France) (*n* = 28) were used. Animal studies were approved by the animal ethics committee of CIC biomaGUNE and local authorities and were conducted in accordance with the Directives of the European Union on animal ethics and welfare. Transient focal ischemia was produced by a 90-min intraluminal occlusion of the middle cerebral artery (MCA) followed by reperfusion as described elsewhere [27]. Briefly, rats were anesthetized with 4% isoflurane in 100% O_2_, and a 2.6-cm length of 4-0 monofilament nylon suture was introduced into the right external carotid artery up to the level where the MCA branches out. Animals were then sutured and placed in their cages with free access to water and food. After 90 min, the animals were re-anesthetized, and the filament was removed to allow reperfusion. Sixteen rats were repeatedly examined with PET before (day 0) and at 1, 3, 7, 14, 21, and 28 days after ischemia to evaluate TSPO binding. Eleven animals were subjected to displacement PET studies at day 7 after the MCA occlusion. The animals studied at day 0 have been considered as the baseline control group. One rat from the displacement study was used to perform ex vivo immunohistochemistry for TSPO receptor expression at day 7 after cerebral ischemia.

### Magnetic resonance imaging

T2-weighted (T_2_W) MRI scans were performed before (day 0) to measure the size of the infarction (*n* = 16) and at 1, 3, 7, 14, 21, and 28 days after the middle cerebral artery occlusion (MCAO) to co-register PET signal data (*n* = 2, one for radiotracer). Before the scans, anesthesia was induced with 4% isoflurane and maintained by 2–2.5% of isoflurane in 100% O_2_ during the scan. Animals were placed into a rat holder compatible with the MRI acquisition systems and maintained normothermia using a water-based heating blanket at 37 °C. MRI experiments were performed on a 7 Tesla Bruker Biospec 70/30 MRI system (Bruker Biospin GmbH, Ettlingen, Germany) and interfaced to an AVANCE III console. The BGA12-S imaging gradient (maximum gradient strength 400 mT/m switchable within 80 μs), an 82-mm-inner-diameter quadrature volume resonator for transmission and surface rat brain coil for the reception were used. T_2_W images were acquired with a rapid acquisition with relaxation enhancement (RARE) sequence with the following parameters: RARE factor 2, TR/TE = 4400/40 ms, FOV = 25 × 25 mm, ACQ Matrix =256 × 256, slice thickness = 1 mm, 2 averages and 24 contiguous slices. Contiguous slices covering all the infarcted volume were acquired and fat suppression was used.

### Magnetic resonance imaging image analysis

MRI (T_2_W) images at 1 day after ischemia were used to calculate the lesion volume. Regions of interest (ROIs) were manually defined using the Open Source software 3D Slicer image analysis software (Version 3.6.3 www.slicer.org) for each rat on the region of increased signal in the ipsilateral hemisphere. The total lesion volume was calculated by summing the area of the infarcted regions of all slices affected by the lesion.

### Radiochemistry

The synthesis of radiotracers *N*, *N*-diethyl-2-(2-(4-(2-fluoroethoxy) phenyl)-5,7-dimethylpyrazolo [1,5-a] pyrimidin-3-yl) acetamide ([^18^F] DPA-714) and 2-(5,7-diethyl-2-(4-(2-fluoroethoxy) phenyl) pyrazolo [1,5-a] pyrimidin-3-yl)-*N*, *N*-diethylacetamide ([^18^F] VUIIS1008) were prepared from their corresponding tosylate precursors according to previously reported procedures using a TRACERlab FX_FN_ synthesis module (GE Healthcare) [25]. Briefly, once transferred into a dedicated (ventilated and lead-shielded) hot cell, [^18^F] fluoride was first trapped on a preconditioned Sep-Pak® Accell Plus QMA Light cartridge (Waters, Milford, MA, USA) and subsequently eluted from the cartridge with a solution of Kryptofix K2.2.2/K_2_CO_3_ in a mixture of water and acetonitrile. After azeotropic drying of the solvent, a solution containing the appropriate tosylate precursor (4.0 mg) in dimethylsulfoxide (0.7 mL) was added, and the mixture was heated at 165 °C for 5 min. The reactor was then cooled at room temperature; the reaction crude was diluted with a mixture of acetonitrile and water (2/1, 3 mL) and purified by HPLC using a Nucleosil 100-7 C18 column (Macherey-Nagel, Düren, Germany) as stationary phase and 0.1 M aqueous ammonium formate solution (pH = 3.9)/acetonitrile (30/70) as the mobile phase at a flow rate of 7 mL/min. The desired fraction (10–11 min for [^18^F] DPA-714, 12–13 min for [^18^F] VUIIS1008) was collected, diluted with water (20 mL), and the radiotracer was retained on a C-18 cartridge (Sep-Pak® Light, Waters, Milford, MA, USA) and further eluted with ethanol (1 mL). The ethanol solution was finally reconstituted with saline solution (9 mL). Filtration through a 0.22 μm filter yielded the final solution, ready for injection. Radiochemical yields (non-decay corrected) were in the range of 8–13% for both [^18^F] DPA-714 and [^18^F] VUIIS1008. Radiochemical purity was consistently higher than 98% at the time of injection. The specific activity for both tracers at injection time was in the range of 175–800 GBq/μmol. Considering the amount of radioactivity injected (ca. 70 MBq/animal), this results in a mass amount injected in the range of 0.04–0.17 μg/animal.

### Positron emission tomography scans and data acquisition

PET scans were repeatedly performed before (day 0) and at 1, 3, 7, 14, 21, and 28 days after reperfusion using a General Electric eXplore Vista CT camera (GE Healthcare). Scans were performed in rats anesthetized with 4% isoflurane and maintained by 2–2.5% of isoflurane in 100% O_2_. The tail vein was catheterized with a 24-gauge catheter for intravenous administration of the radiotracer. Animals were placed into a rat holder compatible with the PET acquisition system and maintained normothermia using a water-based heating blanket. Two groups of animals were subjected to PET scans to assess TSPO expression with [^18^F] VUIIS1008 and [^18^F] DPA-714 at each time point before and after ischemia onset. The radiotracers ([^18^F] VUIIS1008 or [^18^F] DPA-714, ~ 70 MBq) were injected concomitantly with the start of the PET acquisition, and dynamic brain images were acquired (31 frames: 3 × 5, 3 × 10, 3 × 15, 3 × 30, 4 × 60, 4 × 120, 5 × 180, 6 × 300 seconds) in the 400–700 keV energetic window, with a total acquisition time of 60 min. For the displacement studies, unlabeled compounds (VUIIS1008 and DPA-714, 1 mg/Kg) were injected 20 min after the injection of [^18^F] VUIIS1008. After each PET scan, CT acquisitions were also performed (140 μA intensity, 40 kV voltage), providing anatomical information of each animal as well as the attenuation map for the later image reconstruction. Dynamic acquisitions were reconstructed (decay and CT-based attenuation corrected) with filtered back projection (FBP) using a ramp filter with a cutoff frequency of 0.5 mm^−1^.

### Positron emission tomography image analysis

PET images were analyzed using PMOD image analysis software (PMOD Technologies Ltd., Zürich, Switzerland). To verify the anatomical location of the signal, PET images were co-registered to the anatomical data of a MRI rat brain template. Two types of volumes of interest (VOIs) were established as follows: (i) A first set of VOIs was defined to study the whole brain PET signal over time. Whole brain VOIs were manually drawn in both the entire ipsilateral and contralateral hemispheres containing the territory irrigated by the middle cerebral artery on slices of a MRI (T_2_W) rat brain template from the PMOD software. (ii) A second set of VOIs was automatically generated in the cortex, striatum, hippocampus, thalamus, and cerebellum by using the regions proposed by the PMOD rat brain template. The last three time frames of the time-activity curve in a steady state were used to calculate the summed PET binding uptake during the last 15 min of acquisition for both radiotracers. PET signal uptake was averaged in each VOI and expressed as a percentage of injected dose per cubic centimeter (%ID/cm^3^).

### Immunohistochemistry

Immunohistochemistry staining was performed at day 7 after reperfusion. The animal was terminally anesthetized and sacrificed by decapitation. The brain was removed, frozen, and cut into 5-μm-thick sections in a cryostat. Sections were fixed in acetone (− 20 °C) during 2 min, washed with phosphate-buffered saline (PBS) and saturated with a solution of bovine serum albumine (BSA) 5%/Tween 0.5% in PBS during 15 min at room temperature, and incubated during 1 h at room temperature with primary antibodies in a solution of BSA (5%)/Tween (0.5%) in PBS. The section was stained for CD11b with mouse anti-rat CD11b (1:300; Serotec, Raleigh, NC, USA) and for TSPO with a rabbit anti-rat TSPO (NP155, 1:1000). Sections were washed (3 × 10 min) in PBS and incubated for 1 h at room temperature with secondary antibodies Alexa Fluor 350 goat anti-rabbit IgG and Alexa Fluor 594 goat anti-mouse IgG (Molecular Probes, Life Technologies, Madrid, Spain, 1:1000) in BSA 5%/Tween 0.5% in PBS, washed again (3 × 10 min) in PBS, and mounted with a prolong antifade kit in slices (Molecular Probes Life Technologies, Madrid). Standardized images acquisition was performed with an Axio Observer Z1 (Zeiss, Le Pecq, France) equipped with a motorized stage.

### Statistical analyses

For PET signal values, the statistical analysis was performed as follows: The percentage of injected dose per cubic centimeter (%ID/cm^3^) for each animal, the brain hemisphere (ipsilateral and contralateral), and the region (cortex, striatum, hippocampus, thalamus, and cerebellum) were calculated at each time point after cerebral ischemia. Values of %ID/cm^3^ within each region and time point following cerebral ischemia were averaged and compared with the averaged baseline control values at days 0 and 7 using a two-way ANOVA followed by Tukey’s multiple comparison tests for post hoc analysis. The ratio of the lesion to the contralateral brain hemispheres of non-displaced and displacement experiments was compared using a one-way ANOVA followed by Tukey’s multiple comparison tests for post hoc analysis. The level of significance was regularly set at *P* < 0.05. Statistical analyses were performed with GraphPad Prism version 6 software.

## Results

**Fig. 1:**
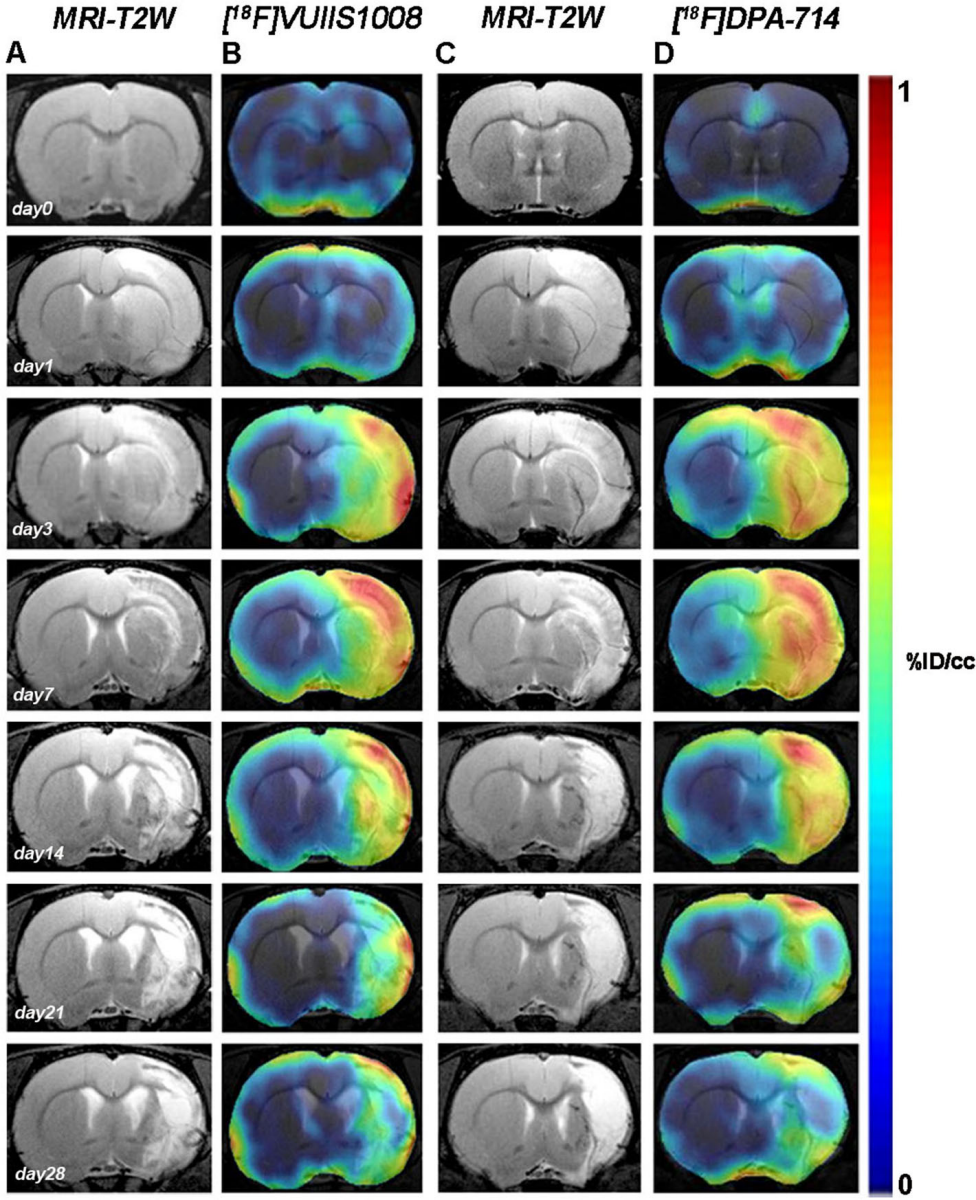
Magnetic resonance imaging (MRI) (T2-weighting (T_2_W)) and positron-emission tomography (PET) images of [^18^F] VUIIS1008 and [^18^F] DPA-714 at control (day 0) and days 1, 3, 7, 14, 21, and 28 after the middle cerebral artery occlusion (MCAO). Serial MRI (T_2_W) (**a**, **c**) and TSPO PET binding (**b**, **d**) images of coronal planes at the level of the lesion are shown. PET images are co-registered with the MRI (T_2_W) of the same animal at different times to localize the PET signal. Images correspond to the lesion evolution of the same animal over time

**Fig. 2:**
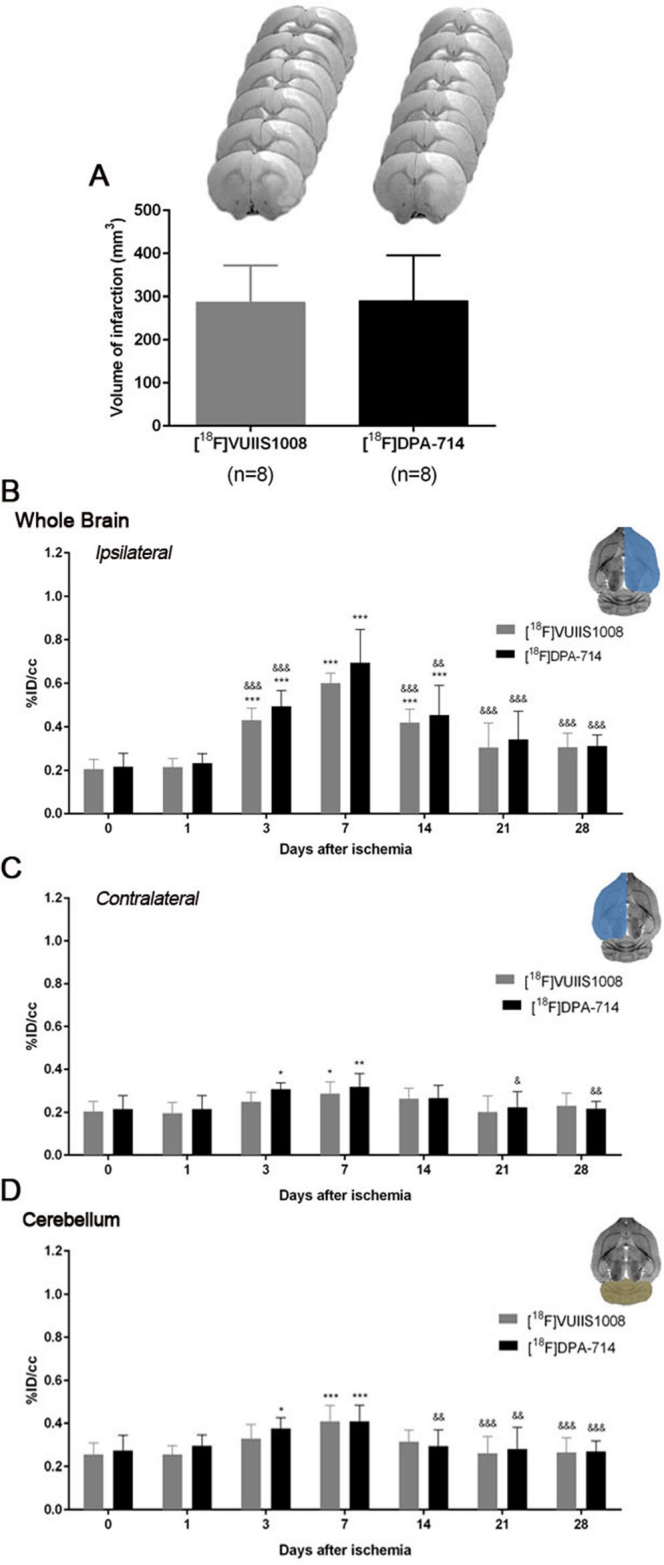
Time course of the progression of the [^18^F] VUIIS1008 and [^18^F] DPA-714 PET signals before and after cerebral ischemia. The infarct size was determined by MRI at 24 h after MCAO (**a**). %ID/cm^3^ (mean ± SD) of [^18^F] VUIIS1008 and [^18^F] DPA-714 was quantified in three VOIs. The entire ipsilateral cerebral hemisphere (**b**), contralateral hemisphere (**c**), and cerebellum (**d**) are shown. The upper right panels of each figure show the selected brain ROIs for the quantification defined on a slice of a MRI (T_2_W) template. Rats (*n* = 8 per group) were repeatedly examined by PET before (day 0) and at 1, 3, 7, 14, 21, and 28 days after ischemia. *P < 0.05, **P < 0.01, and ***P < 0.001 compared with control (day 0) and day 1; ^&^ *P* < 0.05, ^&&^ *P* < 0.01, and ^&&&^ *p* < 0.001 compared with day 7

**Fig. 3:**
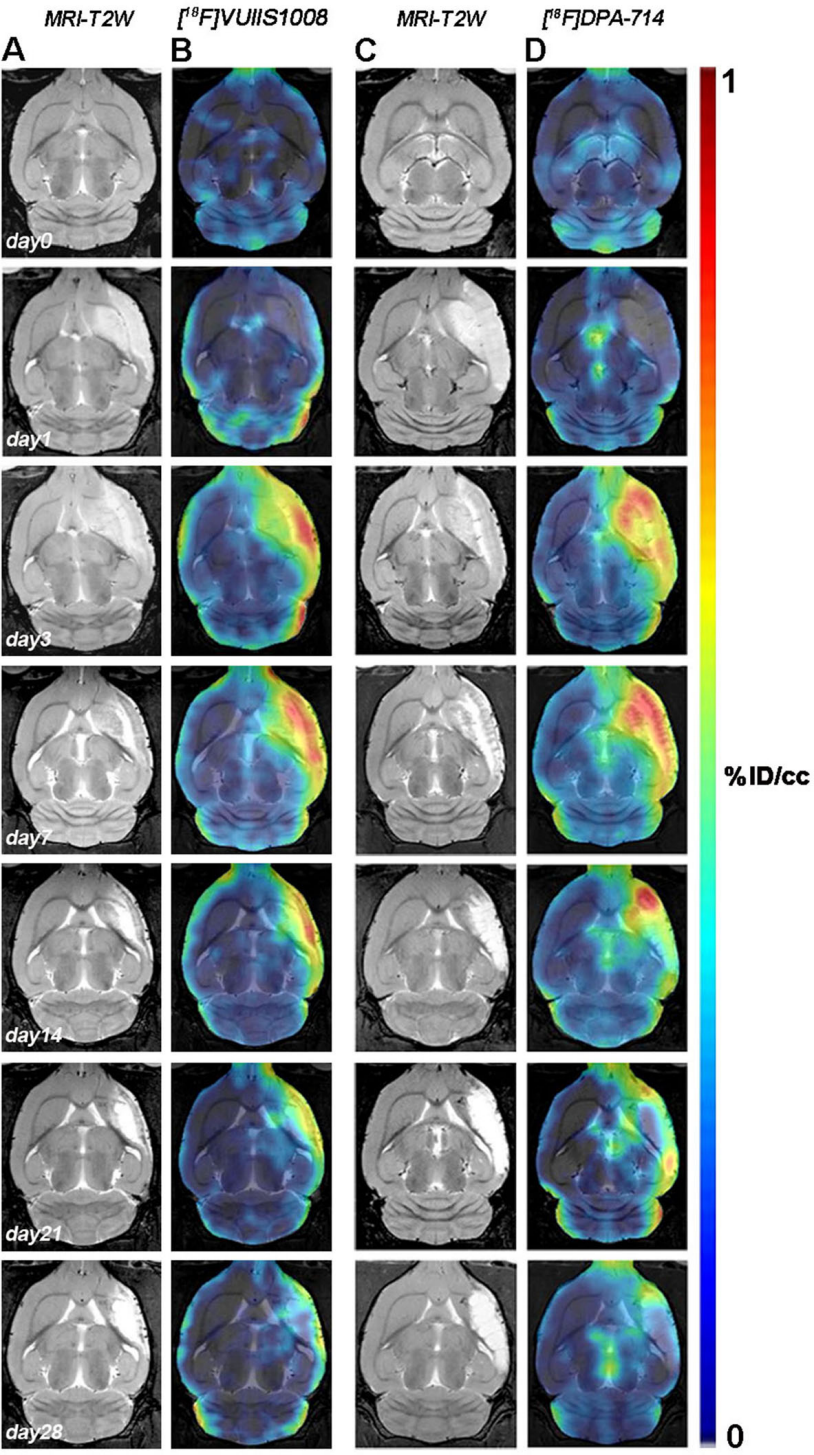
Magnetic resonance imaging (MRI) (T2-weighting (T_2_W)) and positron-emission tomography (PET) images of [^18^F] VUIIS1008 and [^18^F] DPA-714 at control (day 0) and days 1, 3, 7, 14, 21, and 28 after the middle cerebral artery occlusion (MCAO). Serial MRI (T_2_W) (**a**, **c**) and TSPO PET binding (**b**, **d**) images of horizontal planes at the level of the lesion are shown. PET images are co-registered with the MRI (T_2_W) of the same animal at different times to localize the PET signal. Images correspond to the lesion evolution of the same animal over time

Coronal and horizontal T_2_W-MRI images showed the extension and evolution of the cerebral infarction by means of the brain edema at days 0 (control), 1, 3, 7, 14, 21, and 28 after ischemic stroke in rats (Figs. 1 and 3). MRI-T_2_W images were used to co-register PET signal data and to measure the infarct volume at 24 h after the ischemia onset. All rats included in the PET binding time course evaluation for both [^18^F] VUIIS1008 and [^18^F] DPA-714 presented similar infarct volume values that affected the cortical and striatal regions ([^18^F] VUIIS1008 287.43 ± 84.32 mm^3^, *n* = 8; [^18^F] DPA-714 290.58 ± 94.47 mm^3^, *n* = 8) (Fig. 2a). [^18^F] VUIIS1008 and [^18^F] DPA-714 PET signals showed higher uptake in hyperintense lesions on T_2_W-MRI images evidencing the expression of TSPO following cerebral ischemia.

### [^18^F] VUIIS1008 and [^18^F] DPA-714 PET after cerebral ischemia

**Fig. 4:**
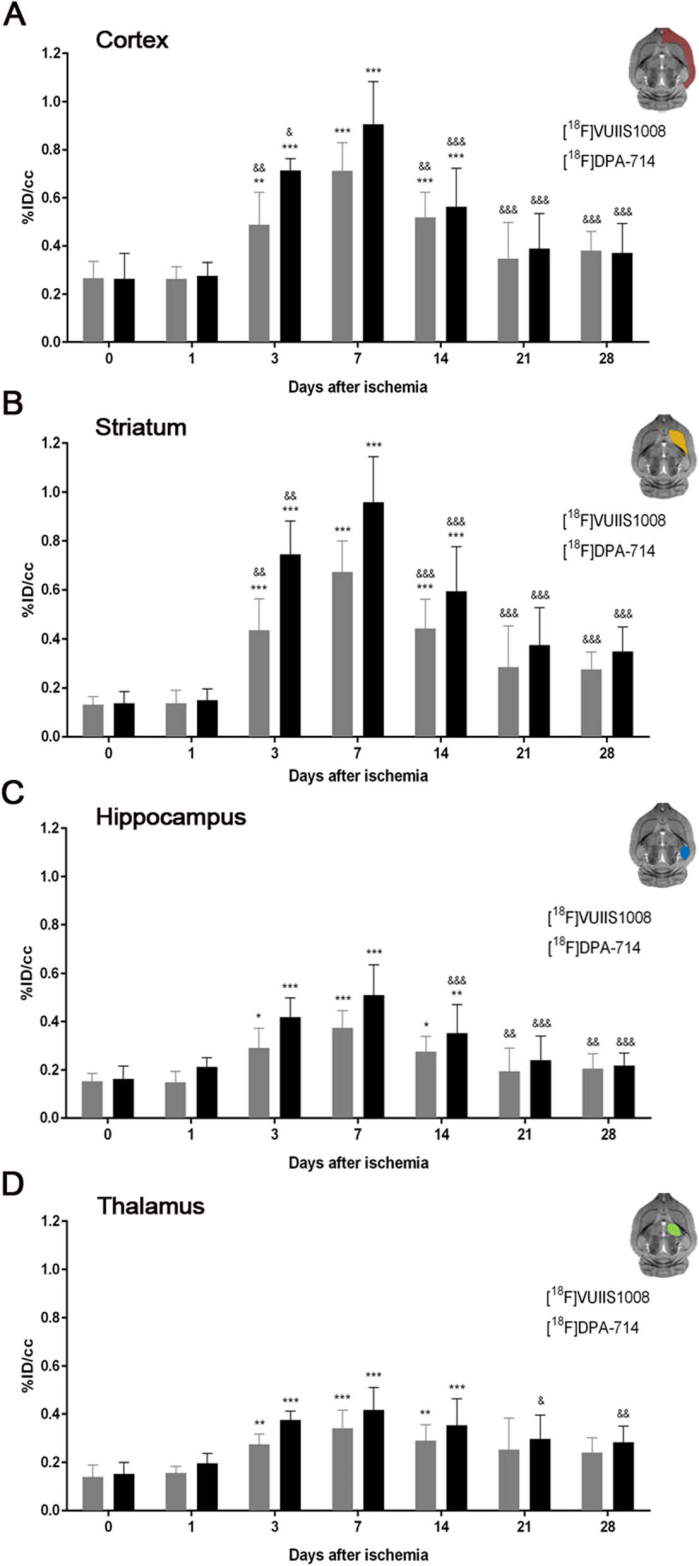
Time course of the progression of the [^18^F] VUIIS1008 and [^18^F] DPA-714 PET signals before and after cerebral ischemia. %ID/cm^3^ (mean ± SD) of [^18^F] VUIIS1008 and [^18^F] DPA-714 was quantified in the ipsilateral cortex (**a**), striatum (**b**), hippocampus (**c**), and thalamus (**d**). The upper right panels of each figure show the selected brain ROIs for the quantification defined on a slice of a MRI (T_2_W) template. Rats (*n* = 8 per group) were repeatedly examined by PET before (day 0) and at 1, 3, 7, 14, 21, and 28 after ischemia. *p < 0.05, **p < 0.01, and ***p < 0.001 compared with control (day 0) and day 1; ^&^ *p* < 0.05, ^&&^ *p* < 0.01, and ^&&&^ *p* < 0.001 compared with day 7

The coronal and horizontal brain images shown in Figs. 1 and 3, illustrate the evolution of the [^18^F] VUIIS1008 and [^18^F] DPA-714-PET signals in ischemic animals at day 0 (control) and at 1, 3, 7, 14, 21, and 28 days after reperfusion. Quantification of the images provided information related to the time-course activity of [^18^F] VUIIS1008 and [^18^F] DPA-714 in both the ipsilateral and contralateral brain whole hemispheres, cerebellum (Fig. 2, *n* = 8), and particularly in the ipsilateral cortex, striatum, hippocampus, and thalamus (Fig. 4, *n* = 8) at the different time points following MCAO. In the ipsilateral whole brain, the PET signal for [^18^F] VUIIS1008 and [^18^F] DPA-714 showed similar low signal intensity before (day 0) and at day 1, followed by a PET signal increase from days 3 to 14 after ischemia (*P* < 0.001, Fig. 2b). In fact, the highest PET uptake signal for both [^18^F] VUIIS1008 and [^18^F] DPA-714 was observed at day 7 compared to days 3, 14, 21, and 28 after MCAO (*P* < 0.01 and *P* < 0.001, Fig. 2b). Subsequently, the PET signal showed a progressive decline from days 14 to 28. Moreover, the [^18^F] DPA-714 uptake signal displayed a mild increase from days 3 to 21 after ischemia compared to the [^18^F] VUIIS1008 PET signal in the ipsilateral hemisphere. In the contralateral whole brain, [^18^F] VUIIS1008 and [^18^F] DPA-714 PET uptake at day 1 showed similar value controls followed by an increase at days 3 and 7 after ischemia (*P* < 0.05; *P* < 0.01, Fig. 2c). In addition, [^18^F] DPA-714 signal showed a higher increase at day 7 compared to days 21 and 28 (*P* < 0.05; *P* < 0.01, Fig. 2c). The cerebellum also showed a PET signal increase at day 7 for both radiotracers compared to different days following ischemia (*P* < 0.05; *P* < 0.001, Fig. 2d). In addition, this increase was followed by a slight decline from days 14 to onwards (*P* < 0.01; *P* < 0.001, Fig. 2d).

The main brain areas affected in this animal model of cerebral ischemia (MCAO) are the cortex and striatum. PET signal uptake for both radiotracers displayed similar distribution pattern over time in the cortex and striatum; nevertheless, [^18^F] DPA-714 showed a mild uptake increase in comparison to [^18^F] VUIIS1008 (Fig. 3a, b). The ipsilateral cortex and striatum displayed a PET signal increase from days 3 to 14 (*P* < 0.001, Fig. 4a, b). Moreover, the PET signal for both radiotracers peaked at day 7 in comparison to different days after MCAO (*P* < 0.05; *P* < 0.01; *P* < 0.001, Fig. 4a, b).

Neighboring regions of the brain lesion such as the hippocampus and the thalamus exhibited PET signal uptake increase for both [^18^F] VUIIS1008 and [^18^F] DPA-714. In fact, the hippocampus showed a higher PET signal uptake compared to the thalamus due to its closer location to the cerebral infarction. The hippocampus and thalamus showed a progressive increase during the first week after reperfusion (*P* < 0.05; *P* < 0.01; *P* < 0.001, Fig. 4c, d), followed by a decline after day 7 (*P* < 0.05; *P* < 0.01; *P* < 0.001, Fig. 4c, d).

### [^18^F] VUIIS1008 PET displacement studies after MCAO

**Fig. 5:**
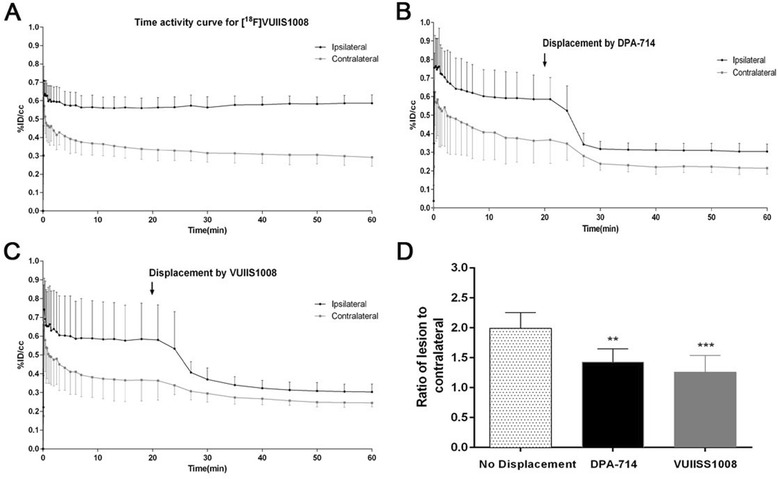
Time-activity curves for [^18^F] VUIIS1008 and displacements by an excess of 1 mg/Kg of either DPA-714 or VUIIS1008 at day 7 after ischemia onset. The activity curve of the VOI placed on the ipsilateral (lesion) or on the contralateral hemisphere of rats injected with [^18^F] VUIIS1008 (*n* = 8) (**a**) are shown. Displacement experiments: [^18^F] VUIIS1008 followed by DPA-714 (*n* = 5) (**b**) and [^18^F] VUIIS1008 followed by VUIIS1008 (*n* = 6) (**c**). **d** Corresponding ratios of the lesion to the contralateral brain hemispheres of non-displaced and displacement experiments at 60 min. Data are expressed as %ID/cm^3^ ratios of the lesion to the contralateral brain hemisphere. Arrows indicate the time of displacement at 20 min after radiotracer injection

**Fig. 6:**
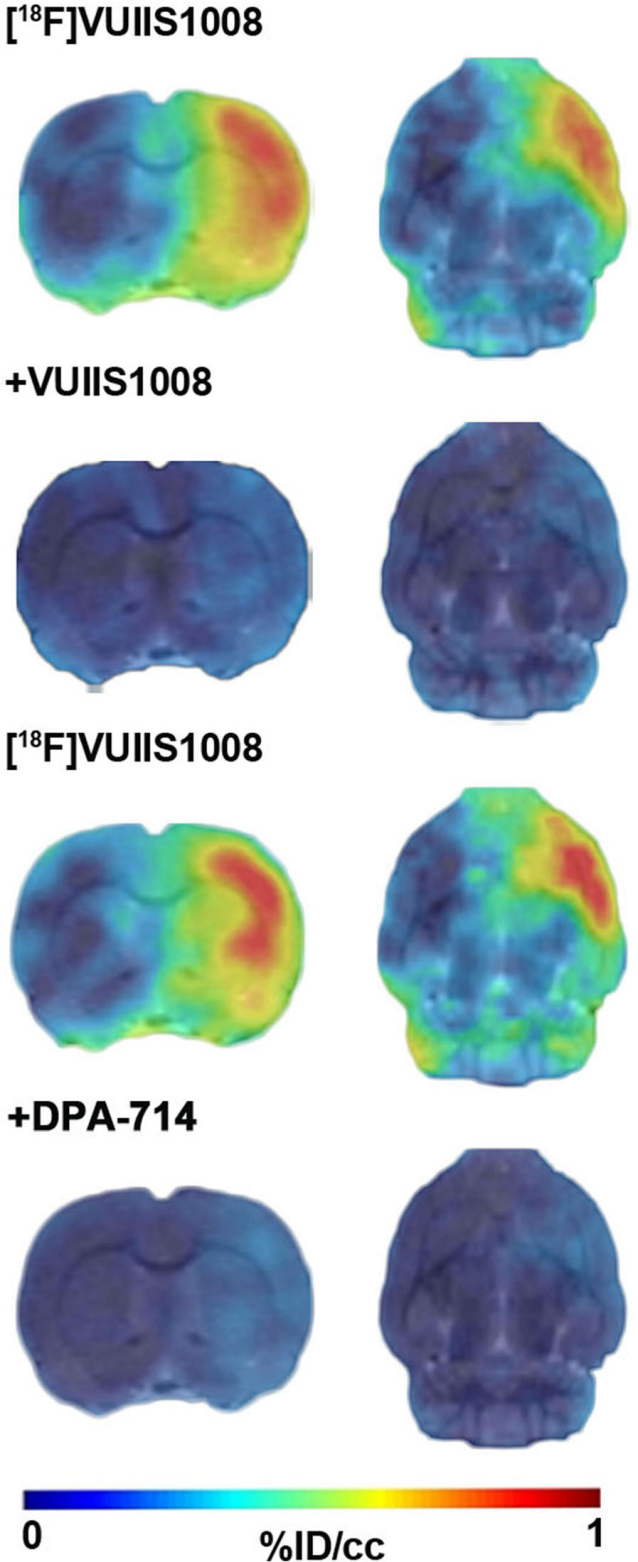
Normalized PET images of [^18^F] VUIIS1008 and displacements by VUIIS1008 and DPA-714. PET images (ID%/cc) of the coronal and horizontal planes at the level of the lesion from left to right. Summed images of [^18^F] VUIIS1008 before displacement (0 to 20 min) and after displacement (40 to 60 min) by VUIIS1008 (upper row) and by DPA-714 (lower row). PET images were co-registered with a rat brain atlas for illustration of anatomical regions

The time-activity curve (TAC) generated in the ischemic cerebral hemisphere at day 7 after brain ischemia showed that [^18^F] VUIIS1008 uptake reached a peak of radioactivity a few minutes after bolus injection and remained constant from 10 to 60 min. In the contralateral hemisphere, the uptake showed a peak of radioactivity during the first minutes followed by a fast washout (Fig. 5a). Displacement studies were performed by injecting an excess (1 mg/Kg) of DPA-714 or VUIIS1008 20 min after the radiotracer injection. Ten to 15 min after the injection of the cold compound, the radioactivity concentration in the ischemic area decreased to the concentration levels of the contralateral area (Fig. 5b, c). Likewise, the ratios of the lesion to the contralateral brain hemisphere showed a significant decrease in the PET uptake signal after displacement by DPA-714 and VUIIS1008 7 days after ischemia (*P* < 0.01; *P* < 0.001, Fig. 5d). In addition, displacement by VUIIS1008 showed a mild decrease compared to the displacement achieved by DPA-714 (Fig. 5d). [^18^F] VUIIS1008 PET images showed the PET signal in the lesion before the displacement (0–20 min) and the [^18^F] VUIIS1008 uptake decrease after displacement with VUIIS1008 and DPA-714 (40–60 min) (Fig. 6).

### Microglial/macrophage expression of TSPO after ischemia

**Fig. 7:**
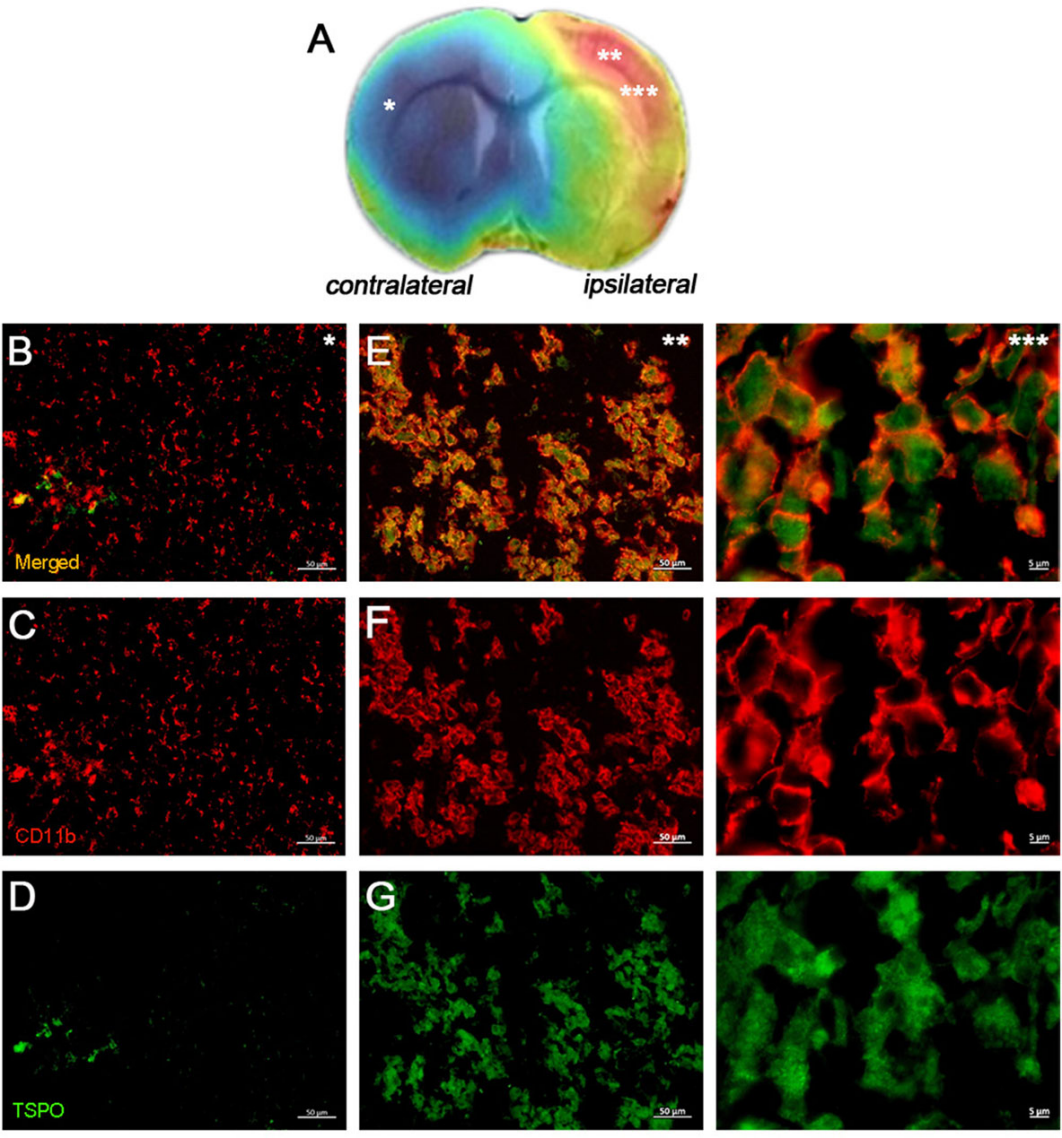
Immunofluorescent labeling of CD11b (red) and TSPO (green) in the contralateral and the ischemic area shown as two channels. The data show the TSPO expression in microglial cells at day 7 after cerebral ischemia. **a** Regions selected for immunohistochemistry studies (*, **, and ***) in a PET image of [^18^F] VUIIS1008 co-registered with the MRI-T_2_W of the same animal at day 7 after MCAO. **b**, **e** Merged images for the two immunofluorescent antibodies in the contralateral and ipsilateral brain hemispheres at different magnifications (*20× and **100×). **f** CD11b-reactive microglia/macrophages are increased in the lesion. **g** TSPO overexpression corresponds to the CD11b-reactive cells. Scale bars *50 μm and **5 μm

The evaluation of TSPO expression was assessed with immunohistochemistry at different magnifications in the contralateral hemisphere and the core of the infarction, as represented in the co-registered [^18^F] VUIIS1008 PET-MRI T_2_W image at day 7 after cerebral ischemia (20×*, 20×** and 100×**, Fig. 7a). In the contralateral hemisphere, immunofluorescence staining showed the low expression of TSPO in few activated microglial cells (Fig. 7b); unlike the core of the lesion, TSPO showed an overexpression of TSPO in activated microglia/infiltrated macrophages at different magnifications (Merged, Fig. 7e). Cells with the morphology of amoeboid reactive microglia/macrophage showed intense CD11b immunoreactivity in the ischemic lesion (in red; Fig. 7f). Subsequently, the overreactivity of microglia co-localized with the expression of TSPO at day 7 after ischemia (in green, Fig. 7g).

## Discussion

PET imaging of TSPO has widely grown over the last two decades to evaluate the role of neuroinflammation in the central nervous system diseases and to assess novel anti-inflammatory therapeutic strategies [21, 28–32]. Moreover, several PET radioligands have been evaluated as markers of microglial neuroinflammation after stroke in both human and animal models [29]. Among them, [^18^F] DPA-714 has been considered as an excellent candidate for imaging neuroinflammation after cerebral ischemia in rodents [3, 24, 33, 34]. Alternatively, [^18^F] VUIIS1008, a novel radiotracer [^18^F] VUIIS1008 with an optimized pyrazolopyrimidine scaffold and approximately 36-fold enhancement in affinity compared to its analog [^18^F] DPA-714, has been recently developed [25]. Because of this, we have assessed the evaluation of both PET radiotracers during the first month following cerebral ischemia in rats, in combination with magnetic resonance imaging (MRI) and immunohistochemistry.

### [^18^F] VUIIS1008 and [^18^F] DPA-714 PET after cerebral ischemia

[^18^F] VUIIS1008 has been previously evaluated as a novel radiotracer for PET imaging of glioma in rats [25, 26]. In these studies, the authors showed that [^18^F] VUIIS1008 exhibited a rapid uptake in TSPO-rich tissues and suggested that this radiotracer might improve tumor detection, particularly for those tumors expressing modest levels of TSPO [26]. Likewise, [^18^F] VUIIS1008 displayed a higher tumor-to-background ratio and binding potential compared to [^18^F] DPA-714 suggesting its promising properties for cancer imaging [26]. Nevertheless, the suitability of this radiotracer still needed to be confirmed in other animal models of neuroinflammation such as cerebral ischemia. Thus, the present study has tackled the unprecedented evaluation of [^18^F] VUIIS1008 as a potential biomarker for in vivo PET imaging of neuroinflammation, particularly in a rat model of transient ischemia.

Cerebral ischemia leads to an overexpression of TSPO which has been considered as a sensitive marker related to the size of the brain lesion and clinical outcome [35]. For this reason and to avoid bias, rats with similar brain infarct volumes measured with MRI-T_2_W at 24 h after reperfusion were included in the PET studies with [^18^F] VUIIS1008 and [^18^F] DPA-714.

Previous in vivo PET imaging of TSPO with [^18^F] DPA-714 showed low levels of the PET signal before the ischemia onset evidencing the low expression of TSPO in the healthy cerebral tissue [3, 33]. In the present study, both [^18^F] VUIIS1008 and [^18^F] DPA-714 radiotracers showed non-significant binding differences in the healthy brain parenchyma before the induction of the MCAO (0. 204%ID/cm^3^ vs. 0.215%ID/cm^3^, respectively). These results stand in agreement with those observed by Tang and collaborators, who described similar tracer uptake values in the healthy brain at 90 min after injection of [^18^F] VUIIS1008 and [^18^F] DPA-714. Despite this, by applying compartmental modeling, the same authors observed a lower influx-to-efflux parameter ratio (k1/k2) and volume of distribution (Vt) for [^18^F] VUIIS1008 compared to [^18^F] DPA-714, but similar binding potential values (k3/k4) [26].

Here, we have observed that following cerebral ischemia, both radiotracers displayed significant accumulation in the injured brain regions at different days after reperfusion (Figs. 1 and 3). In the ischemic hemisphere and particularly in the specific brain regions evaluated (cortex, striatum, hippocampus, and thalamus), PET signal uptake showed the same pattern observed recently by Domercq and colleagues, a progressive [^18^F] DPA-714 uptake increase during the first week followed by a binding gradual decrease afterwards [33] (Figs. 2 and 4). Moreover, we have observed that [^18^F] DPA-714 displayed a mild signal increase from day 3 to day 21 after ischemia in relation to [^18^F] VUIIS1008. In fact, the [^18^F] DPA-714 uptake increase observed at these time points was concomitant with the overexpression of TSPO, particularly at day 7 after ischemia as shown using immunohistochemistry for TSPO (Fig. 7). On the contrary, both radiotracers displayed similar PET uptake values in the ischemic tissue at days 1 and 28 and in non-ischemic brain regions (contralateral hemisphere and cerebellum), scenarios with poor TSPO expression. Therefore, these findings might suggest that [^18^F] VUIIS1008 did not exhibit a greater performance than [^18^F] DPA-714 for imaging high levels of TSPO expression after experimental stroke.

### [^18^F] VUIIS1008 PET displacement studies after cerebral ischemia

At day 7 after ischemia, the time-activity curve for [^18^F] VUIIS1008 showed a fast increased uptake in the ischemic hemisphere that reached a peak 10 min after tracer injection and was maintained at a plateau level during the following 50 min. These findings are in agreement with those displayed by [^18^F] DPA-714 at day 7 after MCAO in rats [3]. In contrast, the contralateral hemisphere showed lower [^18^F] VUIIS1008 uptake because of the low presence of TSPO in healthy tissue. Actually, the contralateral binding was displaced by cold VUIIS1008 and DPA-714 supporting the view that the modest and transient increase of [^18^F] VUIIS1008 binding is TSPO specific (Figs. 5 and 6). Likewise, [^18^F] VUIIS1008 binding was rapidly displaced from the injured hemisphere by an excess of the corresponding unlabeled compound, where, after 10 min, its uptake was close to that in the non-ischemic area. The target-to-background ratio after the displacement by DPA-714 and VUIIS1008 was 1.4 and 1.2, respectively, showing a slight displacement increase performed by VUIIS1008 in relation to DPA-714. In fact, this situation is mainly caused by a more effective displacement of both specific and non-specific TSPO binding of [^18^F] VUIIS1008 by VUIIS1008 in relation to DPA-714. In addition, the fact that [^18^F] DPA-714 could successfully displace [^18^F] VUIIS1008 supports the specificity of this latter to bind to TSPO in vivo following cerebral ischemia. Finally, the radioactivity remaining after displacement studies observed in the lesion compared to the contralateral hemisphere for both radiotracers might be due to the increase of the radiotracer influx/efflux ratio (k1/k2) as a consequence of the blood-brain barrier disruption following cerebral ischemia.

## Conclusions

We report here the PET imaging of TSPO with both [^18^F] VUIIS1008 and [^18^F] DPA-714 in a rat model of cerebral ischemia. Our results confirmed the progressive binding increase of [^18^F] VUIIS1008 in the ischemic hemisphere during the first week after cerebral ischemia, followed by a decline later on. These findings are consistent with the PET signal uptake pattern of [^18^F] DPA-714, a well-known radiotracer for TSPO, and with the expression dynamics of this transporter after cerebral ischemia. Therefore, these results provide novel information about the feasibility of [^18^F] VUIIS1008 to monitor neuroinflammation following neurological diseases such as ischemic stroke.
